# Surface Symphony: Orchestrating DPPC/DOPC Monolayer Behavior

**DOI:** 10.1002/jemt.70066

**Published:** 2025-08-27

**Authors:** Wisnu Arfian A. Sudjarwo, Jose L. Toca‐Herrera

**Affiliations:** ^1^ Institut für Biophysik, Universität für Bodenkultur Wien (BOKU) Vienna Austria

**Keywords:** atomic force microscopy, DOPC, DPPC, Langmuir monolayer

## Abstract

This study investigates the impact of environmental factors and lipid composition on the structural and morphological properties of DPPC/DOPC (1,2‐Dipalmitoyl‐sn‐glycero‐3‐phosphocholine/1,2‐dioleoyl‐sn‐glycero‐3‐phosphocholine) monolayers. Temperature plays a significant role in DPPC monolayer behavior; at 15°C and 20°C, DPPC exhibits liquid expanded–liquid condensed coexistence, while at 25°C, this coexistence region disappears. Divalent ions (CaCl_2_) enhance more compact and uniform DPPC packing compared to monovalent ions (KCl), reducing mean area per molecule. The incorporation of DOPC into DPPC at 20°C increases the monolayer fluidity, disrupting the DPPC domain formation. For all systems, increasing surface pressure improves surface coverage and transforms micro domains into compact and continuous films at higher pressures. These findings highlight the critical roles of ionic interactions, temperature, and lipid composition in modulating lipid monolayer properties.

## Graphical abstract

Langmuir trough and atomic force microscopy reveals the physical nature of DPPC and DOPC mixed monolayers.

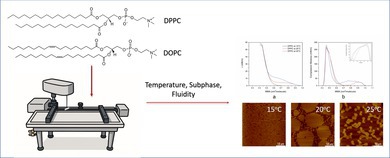

Summary

- Visualization of phase transitions in DPPC‐DOPC mixtures under different thermodynamic conditions.
- AFM imaging reveals how lipid organization is influenced by subphase composition, temperature, and lipid mixtures.
- Monolayer thickness variations are assessed using the scratching method and force‐distance curve analysis.

## 1 Introduction

The molecular architecture and dynamic behavior of lipid membranes are critical to their biological functions, which influence the processes such as signal transduction, vesicle formation, and membrane protein (Casares et al. 2019; Groves and Kuriyan 2010). Among the lipids forming these membranes, dipalmitoylphosphatidylcholine (DPPC) has emerged as a quintessential model due to its well‐defined phase transitions, stability, and relevance in both physiological and synthetic contexts. DPPC is widely studied not only because of its abundance in cell membranes and synthetic lipid systems but also because it provides a benchmark for understanding the physical principles that govern membrane organization and fluidity (Kaviratna and Banerjee 2009; Nagle et al. 2019; Tonks et al. 2002).

The study of DPPC monolayers, particularly using the Langmuir trough, has been crucial in understanding how environmental factors affect membrane structure. The Langmuir–Blodgett (LB) technique offers precise control over lipid packing by manipulating surface pressure (Rojewska et al. 2021; Sasabe and Wada 1989). Typical surface pressure‐area (π‐A) isotherms contribute to some extent about the information regarding macroscopic structures; however, the arrangement of the order at the micro and nanoscale requires advanced high‐resolution experimental techniques. This task can be performed with the help of atomic force microscopy (AFM) which provides in situ imaging of mono‐ or bilayers of lipids. AFM is a powerful tool that captures high‐resolution images of lipid monolayers, which reveal intricate details such as domain formation, phase separations, and surface roughness. Furthermore, it can reveal topography and phase domains with exceptional detail (Robinson et al. 2023; Tharad et al. 2019, 2020). When combined with AFM, the LB technique enables researchers to directly observe the morphology and structural changes of lipid films under different conditions. This integration offers valuable insights into how external factors influence the behavior of lipid monolayers (Balashev et al. 2007; García‐Arribas et al. 2021).

Unlike other imaging techniques, atomic force microscopy (AFM) offers real‐time visualization of monolayer structures without the need for labels, allowing for the observation of micro‐ and nanoscale phase transitions and morphological changes in these monolayers (Henderson et al. 2024; Picas et al. 2012). Consequently, AFM images can provide visual evidence of molecular packing and lipid organization in response to surface compression.

Beyond surface pressure, various experimental factors like subphase composition and temperature can significantly influence the physical properties of monolayers. For example, ions can adjust electrostatic interactions, affecting the formation of condensed or expanded domains (Friedman 2018; Petelska and Naumowicz 2017; Wilke and Maggio 2011). Temperature changes can induce phase transitions, impacting lipid mobility and packing, which can be observed as distinct morphological changes in AFM images (Frey and Lee 2007; Zuo et al. 2016). The mixing of saturated and unsaturated lipids adds further complexity to the monolayer structure, reflecting alterations in lipid domain formation, packing density, and fluidity (Alwarawrah et al. 2010; Guzmán et al. 2012; Leeb and Maibaum 2018; Olżyńska et al. 2016; Qiao et al. 2015; Regen 2022; Sabatini et al. 2008; Zhu et al. 2021).

This study extends previous findings on the thermodynamic properties of lipid monolayers using Langmuir technique and Atomic Force Microscopy (AFM) to provide a comprehensive understanding of how thermodynamic factors affect the visual appearance and surface dynamics of DPPC/DOPC monolayers. We specifically examined the impact of subphase composition, surface pressure, temperature, and lipid mixtures on lipid organization and phase transitions. Although DPPC and DOPC have been widely studied, particularly with the Langmuir–Blodgett technique, our findings offer new insights into how these factors influence the physical and visual phase transitions of DPPC monolayers in greater detail. The combination of Langmuir and AFM techniques offers new perspectives for elucidating these influences more clearly. Additionally, we demonstrated that DPPC and DOPC monolayers exhibit distinct thicknesses, highlighting their differences in the liquid‐condensed and liquid‐expanded phases. These findings have significant implications for fundamental biophysics and the advancement of membrane model development.

## 2 Material and Method

### 2.1 Material

1,2‐Dipalmitoyl‐sn‐glycero‐3‐phosphocholine (DPPC) and 1,2‐dioleoyl‐sn‐glycero‐3‐phosphocholine (DOPC) were sourced from Avanti Polar Lipids (USA). Methanol, chloroform, CaCl_2_·2H_2_O, and KCl were obtained from Merck (Germany). Milli‐Q water was produced using a Millipak Gold 0.22 μm filter, achieving a final resistivity of 18.2 MΩ cm. All lipids were prepared at a concentration of 2 mM in a methanol: chloroform mixture (1:4, v/v) and stored at −20°C. Aqueous solutions of KCl and CaCl_2_ were prepared at a concentration of 10 mM.

### 2.2 Preparation Prior to Measurement

The Langmuir trough (KSV NIMA‐Finland; area of trough = 24,300 mm^2^) was placed on an antivibration table inside a plexiglass box to prevent any vibration and dust contamination. Before the experiment, the Langmuir trough was cleaned with ethanol and a methanol: chloroform mixture (1:4, v/v), followed by two rinses with Milli‐Q water. Subsequently, 135–140 mL of subphase (water, KCl, or CaCl_2_) was poured into the trough. Two moving barriers were positioned in the trough along with a custom‐made Wilhelmy sensor (made from Whatman filter paper No. 1, width of 10.30 mm). A water bath (MGW Lauda Krüss) was connected to the trough to control the subphase temperature via water circulation. The subphase temperature was maintained at 20°C ± 1°C for all measurements, except for the temperature dependence experiment.

### 2.3 Surface Pressure‐Area (π‐A) Isotherm Measurements

The lipid solution was spread using a 20 μL Hamilton syringe, evenly distributing it on the air‐water interface between the two barriers. The trough was left undisturbed for 10 min to stabilize the system and allow the methanol/chloroform solvent to evaporate. After this stabilization period, observations were conducted using the “Constant Barrier Rate Compression” mode. To ensure reproducibility, each measurement was repeated three times. The compression rate was set to 10 mm/min, based on our previous study (Sudjarwo and Toca‐Herrera 2023). All measurements were conducted at 20°C, except for the temperature dependence experiment.

### 2.4 Compression Modulus

The isotherm compression modulus (*Cs* ^−1^) for the specified film compositions at provided surface pressure (π) was derived from π‐*A* data using Equation (1) (Smaby et al. 1996):

(1) $$Cs^{-1}=-A\left(\frac{\partial \pi }{\partial A}\right)$$

where *A* is the area per molecule at specific surface pressures (*π*). The compression modulus serves as a measure of a lipid monolayer's resilience to compression. A higher compression modulus value indicates greater resistance of the lipid film to deformation, while a lower value suggests the opposite.

### 2.5 AFM Measurements

Before observing the lipid with AFM, we transferred the DPPC monolayers onto mica muscovite (Hrubý et al. 2019) at several discrete surface pressures: 3, 5, 10, 20, and 30 mN/m at 20°C, except for the temperature dependence experiment. Briefly, before lipid deposition, mica was glued onto a silicon wafer and submerged at a tilted angle of about 30° with respect to the air/water interface. To ensure the reliability of our observations, each lipid ratio was transferred twice. Each transfer was repeated three times, and multiple spots were analyzed using AFM imaging.

After lipid transfer, AFM imaging was performed in contact mode (in air) using an SPM Multimode (Veeco Bruker, MA, USA). We used a DNP‐S10 cantilever with a tip radius of 10 nm and a spring constant of 0.12 N/m. The spring constant for each cantilever was calibrated using the thermal tune method, yielding values around 0.1366 N/m with minor deviations (within ±10%). All experiments were performed using tips of the same model to ensure consistency. During observation, the applied force was kept below 1 nN to minimize sample damage. The scan rate was set between 1 and 2 Hz, with an image resolution of 512 × 512 pixels. All images and force‐distance curves were processed using NanoScope Analysis 1.5 software (Bruker, Germany), while surface coverage was analyzed using ImageJ version 1.54f (NIH, USA).

To observe the monolayer thickness, in addition to force‐distance curves and thickness profiling, we scratched the monolayer within an area of 1 × 1 μm. A scan rate of 6–10 Hz and a shear force larger than 2 nN were applied, as estimated based on the lateral deflection of the AFM cantilever and its known spring constant (calculated using the thermal tune method). Please refer to the Supporting Information for an example of the calculation. Scanning was performed 10–15 times for both DPPC and DOPC monolayers at room temperature.

## 3 Results and Discussion

### 3.1 Monolayer Structure at Different Temperatures

**FIGURE 1:**
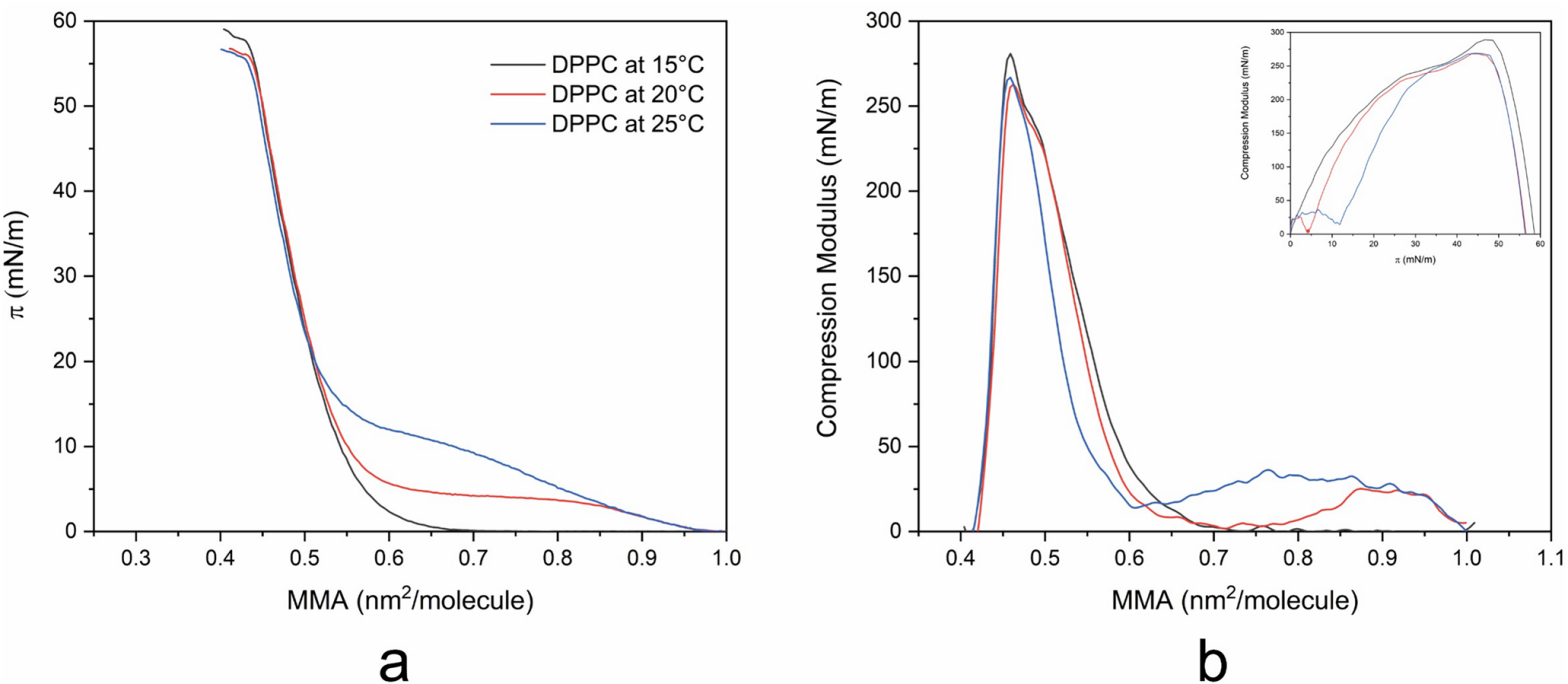
(a) π‐A isotherm curve of DPPC and (b) Compression modulus (Cs^−1^)‐MMA curve of DPPC at three different temperatures: 15°C, 20°C, and 25°C on subphase water. The color of each curve corresponds to the selected temperature. The inset shows the compression modulus (Cs^−1^) versus surface pressure (*π*).

The initial experiment aimed to evaluate the π‐A relationship as a function of temperature. Figure 1 presents the π‐A isotherms and compression modulus (Cs^−1^) versus surface pressure (π) of DPPC monolayers at various selected temperatures.

The results agree with previous findings and other studies (Sudjarwo and Toca‐Herrera 2023; Toimil et al. 2010; Zuo et al. 2016). As shown in Figure 1a, the plateau representing the LE‐LC phase transition of the DPPC monolayer is temperature‐dependent. The plateau shifts to higher surface pressures as the temperature increases (i.e., 20°C and 25°C), and is not noticeable at 15°C. This indicates that above 15°C, all different phases of the DPPC monolayer can be observed: the LE (liquid‐expanded) phase, the coexistence of LE‐LC (liquid‐expanded—liquid‐condensed) phase transition, and the LC (liquid‐condensed) phase. Specifically, at 20°C, the plateau is more pronounced, and flatter compared to that at 25°C. Consequently, the remaining experiments were conducted at 20°C.

Figure 1b illustrates the curve of compression modulus (Cs^−1^) versus mean molecular area (MMA), while the inset shows the curve of compression modulus (Cs^−1^) versus surface pressure (π) at different temperatures. These curves describe the physical state of the monolayer as a function of surface pressure. For instance, the liquid‐expanded phase occurs within a modulus range of 12.5 to 50 mN/m, whereas the liquid‐condensed phase occurs within a range of 10 to 25 mN/m. The results indicate that the liquid‐condensed phase for DPPC occurs at a surface pressure of 8 mN/m, corresponding to an area per molecule of 0.54 nm^2^/molecule at 15°C (Figure 1b inset). At 20°C, the condensed phase is reached at approximately 14 mN/m with an area per molecule of 0.55 nm^2^/molecule, while at 25°C, it appears at a surface pressure of about 20 mN/m with an area per molecule of 0.52 nm^2^/molecule. Therefore, achieving the condensed phase at higher temperatures requires more pressure, although the overall trend of the curves remains similar. Additionally, Figure 1b (inset) shows sharp peak minima for both curves at 20°C and 25°C, corresponding to the observed plateau in Figure 1a. The surface pressure at these peak minima increases with temperature. These sharp minima in the Cs^−1^‐π curve become flatter in the Cs^−1^‐MMA curve.

**FIGURE 2:**
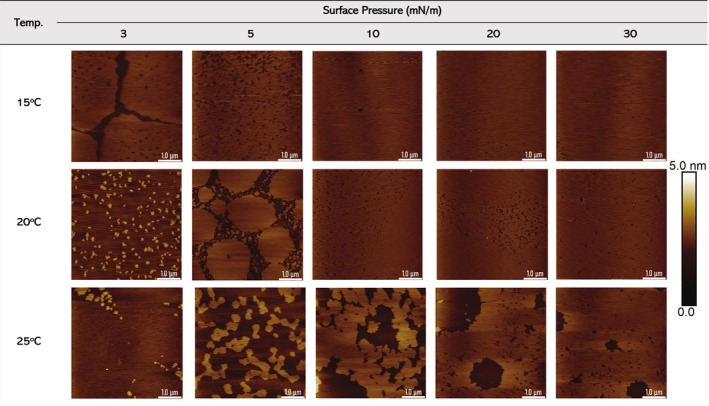
AFM images of DPPC monolayers at three different transfer temperatures: 15°C, 20°C, and 25°C. The scan area was 5 μm × 5 μm. The white scale bar (at the bottom right) corresponds to 1 μm, while the vertical color bar scales from 0 to 5 nm.

Figure 1b shows that the plateau occurs approximately from an MMA of 0.85 nm^2^/molecule to 0.6 nm^2^/molecule at 20°C, and from 0.2 nm^2^/molecule to 0.55 nm^2^/molecule at 25°C. In contrast, at 15°C, neither a sharp minimum in the Cs^−1^‐π curve nor a valley in the Cs^−1^‐MMA curve was observed, indicating the absence of the LE‐LC coexistence region and a high compression modulus. This is further addressed by AFM imaging (Figure 2).

Figure 2 displays AFM images of DPPC monolayers deposited on mica at various surface pressures and temperatures. The bright regions represent lipid domains, with the vertical scale estimating their thickness. Each row corresponds to a different temperature (15°C, 20°C, and 25°C), while each column corresponds to a different surface pressure (ranging from 3 to 30 mN/m). A profile analysis of the lipid monolayers shown in Figure 2 is presented in Figures S1–S3 (Supporting Information).

**FIGURE 3:**
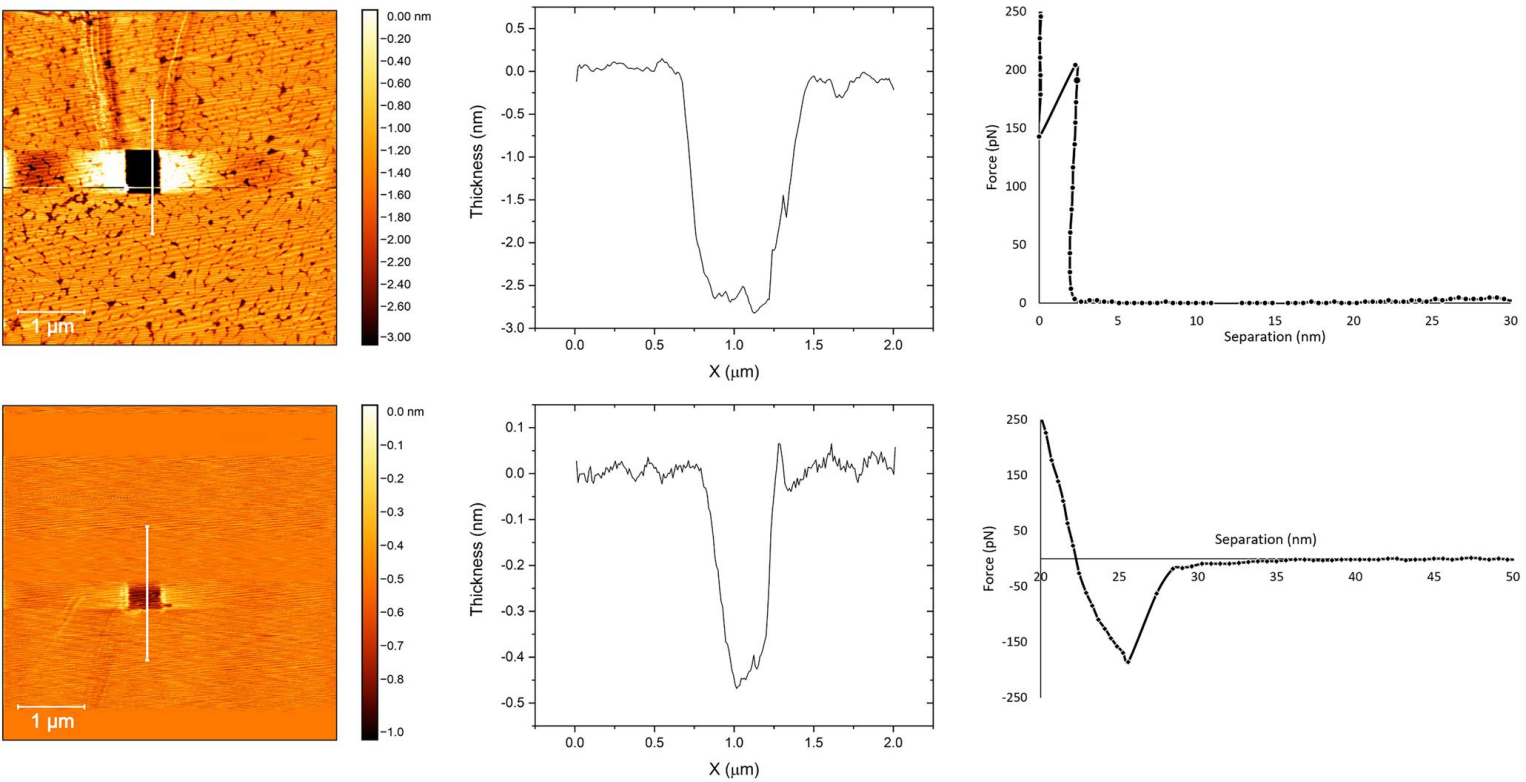
Scratched lipid monolayers, thickness profiles, and force‐distance curves for DPPC (top) and DOPC (bottom). The experiments were conducted at 20°C. The white scale bar (at the bottom left) corresponds to 1 μm.

From the profile analysis, we can distinguish the thickness of the transferred DPPC monolayers particularly between LE and LC phases. The LE–LC coexistence domains exhibit a height difference of approximately 0.97 ± 0.15 nm, while the LC domains (measured from non‐domain (flat) areas) show a thickness of around 1.10 ± 0.13 nm. Although the height contrast between these regions is modest, further investigation using scratching experiments and force–distance (F–D) curves confirmed the actual monolayer thickness in the LC phase. These complementary measurements are presented in Figure 3, supporting the interpretation of the AFM data and confirming successful monolayer transfer.

At 15°C, across surface pressures ranging from 3 to 30 mN/m, the DPPC monolayer exhibits the phase transitions shown in Figure 1. DPPC reaches the condensed phase at low surface pressure. The monolayer demonstrates high initial surface coverage, as shown in Table 1, even at a low pressure of 3 mN/m (87.8%). Additionally, the AFM images show interconnected domains, indicating strong molecular interactions and favorable packing at lower temperatures. As the pressure increases, the monolayer achieves higher molecular order. By 5 mN/m, the surface coverage increases to 92.1%, and at pressures of 10 mN/m and higher, the surface coverage exceeds 95%, resulting in a stable monolayer structure that reaches its final condensed state.

**TABLE 1:** Surface coverage of lipid monolayer at three different transfer temperatures: 15°C, 20°C, and 25°C.

	Surface coverage (%)				
	3 mN/m	5 mN/m	10 mN/m	20 mN/m	30 mN/m
15°C	87.8	92,1	> 95	> 95	> 95
20°C	10.4	76.5	> 95	> 95	> 95
25°C	3.6	29.8	70.7	80.5	94.7

At 20°C, the monolayer exhibits more pronounced structural features. The surface coverage and morphology differ significantly at low pressures. At 3 mN/m, where the liquid‐expanded phase occurs, noticeable small domains appear (Cruz et al. 2005; Zhang et al. 2011), with a surface coverage of about 10.4%. At 5 mN/m, where the coexistence region occurs (Kaganer et al. 1999), these domains merge to form larger ones under higher compression (Zhang et al. 2011; Zuo et al. 2008). However, at higher pressures (10 to 30 mN/m), the monolayer at 20°C begins to acquire a uniform morphology similar to that observed at 15°C, with coverage exceeding 95%. Thus, higher surface pressure leads to more densely packed DPPC molecules, consistent with previous findings (Makyła‐Juzak et al. 2018).

The DPPC monolayer transferred onto mica at 25°C exhibits the most pronounced differences, especially at lower pressures. At 3 mN/m, the coverage is minimal (3.6%), and the images reveal topographical irregularities. By 5 mN/m, the lipid domain coverage remains relatively low at 29.8%, indicating weaker lipid‐lipid interactions probably due to a higher molecular mobility at this temperature. As the surface pressure increases, the domain coverage steadily improves, reaching 70.7% at 10 mN/m and 80.5% at 20 mN/m. Only at 30 mN/m does the monolayer reach a high coverage (94.7%), but the AFM images show more visible defects compared to lower temperatures. This suggests that while the coverage is extensive, structural uniformity may be compromised at this surface pressure.

### 3.2 Monolayer Structure at Different Subphase Conditions

**FIGURE 4:**
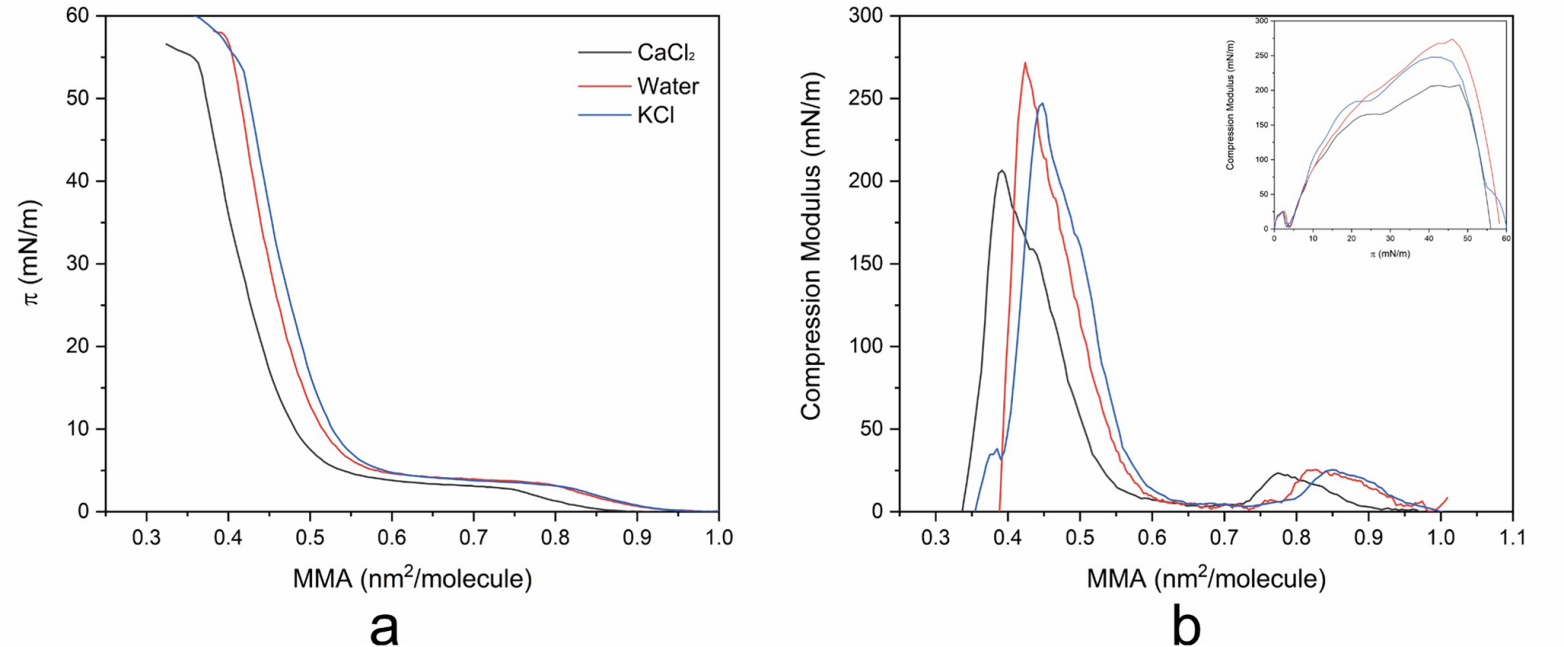
(a) π‐A isotherms of DPPC, and (b) compression modulus (Cs^−1^) versus MMA for three different electrolyte subphases: 10 mM CaCl_2_, water, and 10 mM KCl. The color of each curve corresponds to the specific electrolyte used. The inset shows the compression modulus (Cs^−1^) as a function of surface pressure (π). All measurements were performed at 20°C.

The next step involved examining how electrolytes affect the interaction between lipid molecules. Figure 4 illustrates the π‐A isotherms of DPPC and the compression modulus values as a function of MMA for various electrolyte subphases.

Figure 4a demonstrates the behavior of DPPC monolayer when exposed to CaCl_2_, water, and KCl. The variations in the π‐A curves indicate that each subphase influences the molecular packing and phase behavior of the DPPC monolayer differently. In the CaCl₂ subphase, DPPC exhibits a smaller MMA compared to the water and KCl subphases at the same surface pressure, suggesting that DPPC molecules are more tightly packed in the presence of Ca^2+^ ions. The stronger interactions between Ca^2+^ ions and negatively charged phosphate groups of DPPC likely facilitate closer packing, which leads to a smaller MMA. Ca^2+^ ions are recognized for their capacity to bridge with the phosphate groups of lipid head groups (Adams et al. 2016; Cordomí et al. 2008; Petelska et al. 2010; Zhang et al. 1991), thereby reducing the repulsion between adjacent molecules. Consequently, the DPPC monolayer appears more condensed in the presence of calcium. In contrast, water and KCl result in larger MMAs, indicating a less condensed monolayer. The DPPC monolayer on water exhibits the second‐smallest MMA, whereas the KCl subphase results in the largest MMA, indicating greater repulsion between the lipid molecules. The smaller MMA in the water subphase compared to KCl suggests that pure water promotes more compact molecular packing than KCl. The K^+^ ions do not interact strongly with DPPC molecules, leading to electrostatic repulsion that separates the lipid molecules (Adams et al. 2016; Javanainen et al. 2020; Petelska and Figaszewski 2011).

Figure 4b illustrates the relationship between Cs^−1^ and MMA, as well as between Cs^−1^ and surface pressure (π). Despite having the smallest MMA, the CaCl_2_ subphase exhibits the lowest peak compression modulus. Calcium ions facilitate tighter packing of DPPC molecules, followed by water and KCl. Conversely, the DPPC monolayer on KCl is less compact than on water or CaCl_2_. It is also evident that the plateau in Figure 4a corresponds to the valley in Figure 4b. The plateau for DPPC in the CaCl_2_ subphase begins at an MMA of approximately 0.76 nm^2^/molecule, followed by water at 0.82 nm^2^/molecule and KCl at 0.85 nm^2^/molecule. Regardless of the subphase, the plateau lengths are relatively similar. In the inset of Figure 4b, we observe that all subphases exhibit phase transitions, despite differences in monolayer rigidity. DPPC in all subphases enters the condensed phase at approximately 14 mN/m. The experimental compressibility modulus values reported in this study are consistent with those found in the literature (Adams et al. 2016).

**FIGURE 5:**
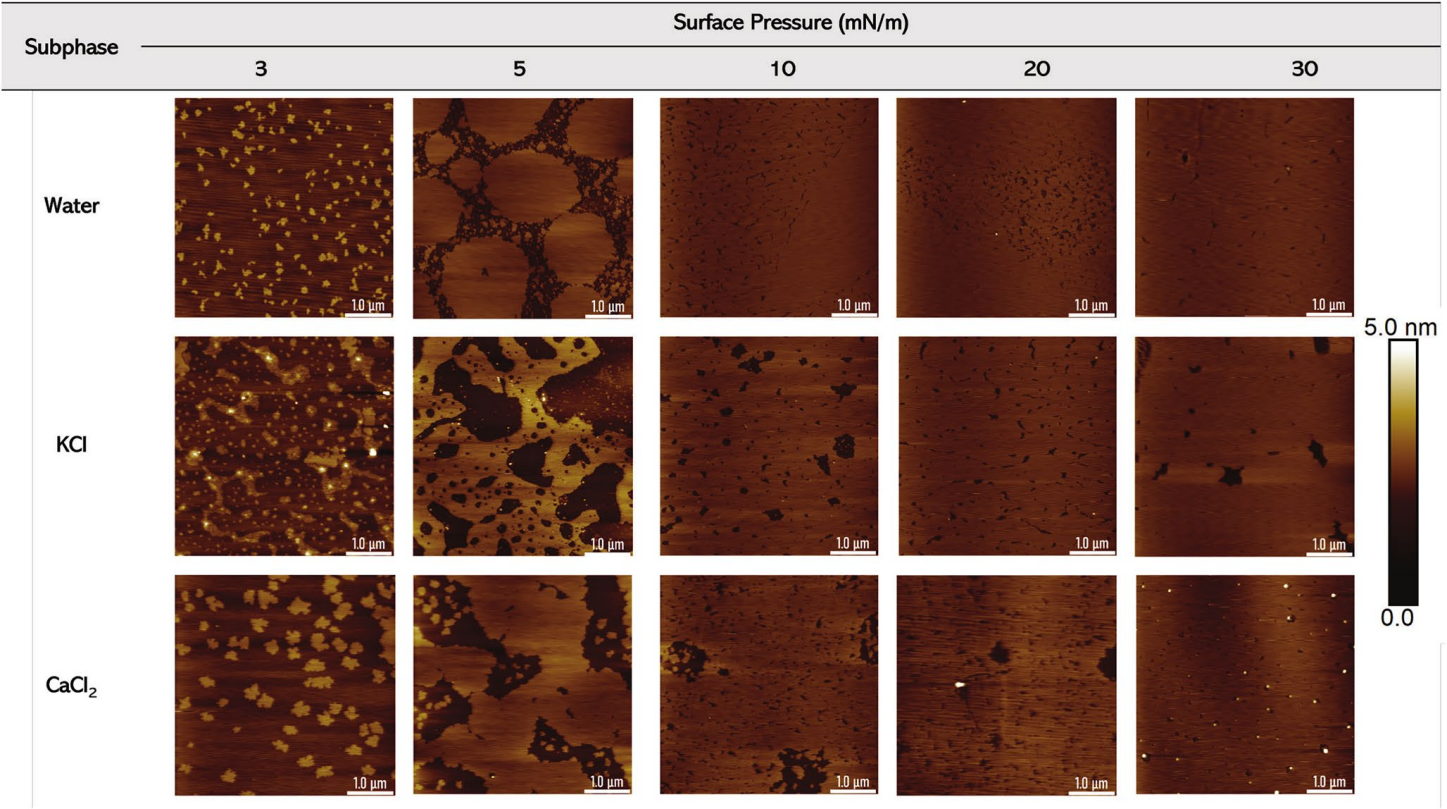
AFM images of DPPC monolayers for three different subphases: 10 mM CaCl₂, water, and 10 mM KCl. The scan area is 5 μm × 5 μm. The white scale bar (at the bottom right) represents 1 μm, while the vertical color bar ranges from 0 to 5 nm.

Figure 5 presents AFM images of transferred DPPC monolayers on different electrolyte subphases at selected surface pressures. At a low surface pressure of 3 mN/m (LE phase), DPPC displays small domains and dispersed aggregates. On water, the monolayer shows sparse domain structures, whereas the KCl and CaCl_2_ subphases exhibit more pronounced aggregation. Table 2, which details surface coverage, supports this observation, with water at 10.4%, KCl at 26.5%, and CaCl_2_ at 24.5%. The ionic subphases promote higher initial surface aggregation compared to pure water. At a surface pressure of 5 mN/m (LE‐LC phase transition), there is a notable increase in lipid surface coverage for all subphases, with values of 76.5% for water, 54.2% for KCl, and 75.9% for CaCl_2_. Additionally, AFM images reveal significant coalescence and growth of DPPC domains, especially on the CaCl_2_ and water subphases, while KCl shows smaller and less connected domains. This indicates that the presence of Ca^2+^ ions facilitates stronger interactions and more compact packing of DPPC molecules compared to K^+^ ions in KCl.

**TABLE 2:** Surface coverage of the lipid monolayer for three different electrolyte subphases: 10 mM CaCl_2_, water, and 10 mM KCl.

	Surface coverage (%)				
	3 mN/m	5 mN/m	10 mN/m	20 mN/m	30 mN/m
Water	10.4	76.5	> 95	> 95	> 95
KCl	26.5	54.2	91.6	> 95	> 95
CaCl_2_	24.5	75.9	93.6	> 95	> 95

At intermediate to high surface pressures (10–20 mN/m), the surface coverage across all subphases exceeds 90%, with water showing over 95% coverage at 10 mN/m. The morphologies at these pressures display uniform and continuous films, indicating the transition of DPPC from a liquid‐expanded to a liquid‐condensed state. While both KCl and CaCl_2_ subphases exhibit similar trends in achieving high coverage, the uniformity of the film is greater in the presence of CaCl_2_, likely due to the strong ion‐lipid interactions that enhance monolayer stability. Finally, at the highest surface pressure of 30 mN/m, all subphases achieve near‐complete surface coverage (> 95%). At this surface pressure, all DPPC monolayers reach the liquid‐condensed phase and show a similar appearance. All ions including K^+^ and Ca^2+^ are squeezed out from DPPC head group regions (Adams et al. 2016; Petelska and Figaszewski 2011; Sovago et al. 2007).

### 3.3 Controlling Monolayer Fluidity by Adding DOPC

**FIGURE 6:**
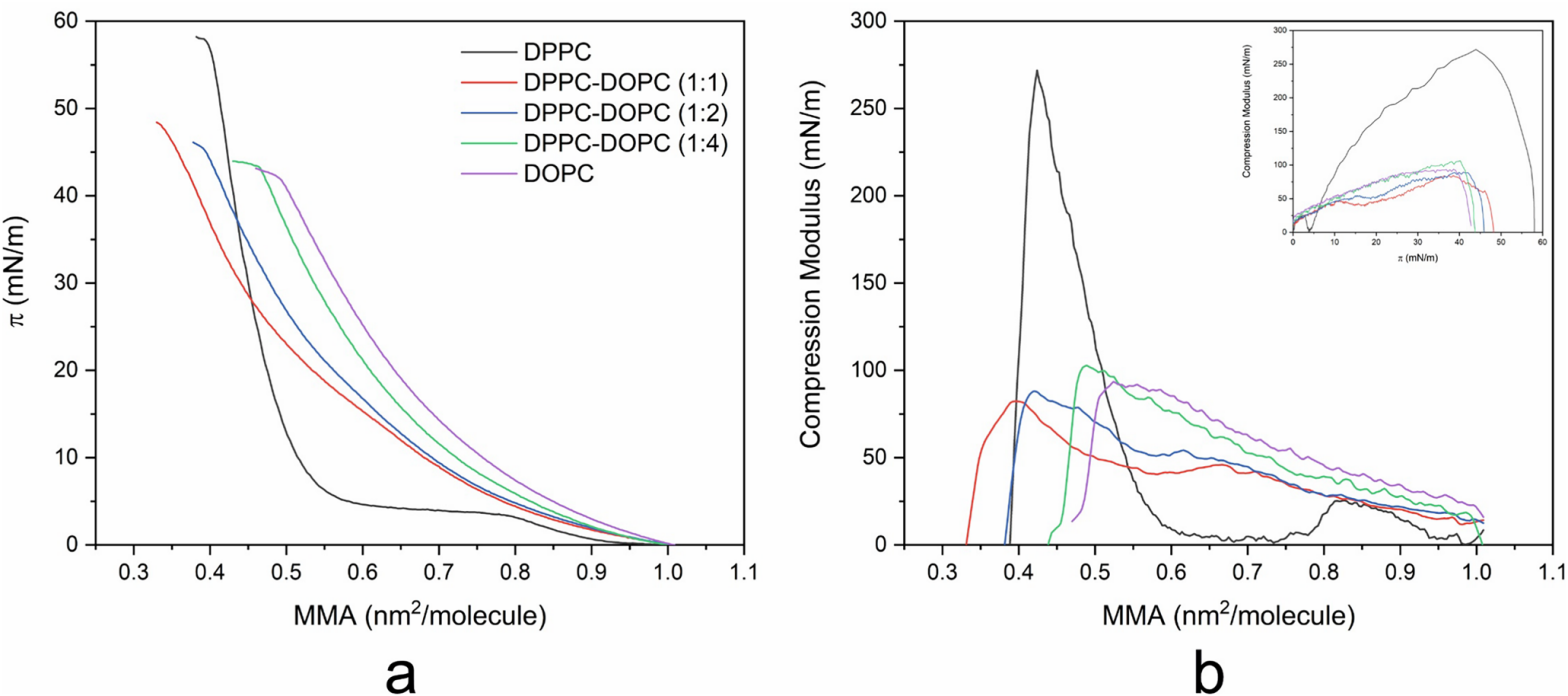
(a) π‐A isotherm of DPPC:DOPC lipid mixtures, (b) compression modulus versus mean molecular area for the lipid mixtures: DPPC, DPPC‐DOPC (1:1), DPPC‐DOPC (1:2), DPPC‐DOPC (1:4), and DOPC. The color of each curve indicates the corresponding lipid mixture. The inset shows the compression modulus versus π. The isotherms were measured at 20°C.

To conclude, we examined how DOPC affects the fluidity of the monolayer. We utilized the following molar mixtures for this study: DPPC, DPPC‐DOPC (1:1), DPPC‐DOPC (1:2), DPPC‐DOPC (1:4), and DOPC. The results of these measurements are presented in Figure 6.

At 20°C, the π–A isotherms in Figure 6a depict the thermodynamic and compression behavior of DPPC, DOPC, and their mixtures. The pure DPPC monolayer (black line) exhibits a collapse pressure around 55 mN/m at an MMA of approximately 0.45 nm^2^/molecule. This relatively high collapse point is due to the interactions between the saturated acyl chains of DPPC, allowing the monolayer to pack densely. In contrast, DOPC, with its unsaturated chains, collapses at a lower pressure of around 45 mN/m and at a larger MMA (~0.55 nm^2^/molecule), indicating that DOPC requires more space and has less tolerance for compression. The isotherms of DPPC:DOPC mixtures (1:1, 1:2, and 1:4) show intermediate collapse pressures that decrease with increasing DOPC concentration, suggesting a gradual shift toward the more fluid, expanded phase typical of DOPC. For example, the DPPC:DOPC (1:1) mixture (red line) collapses at approximately 49 mN/m, whereas the 1:4 mixture (blue line) collapses at around 47 mN/m. This trend indicates a decrease in packing density and an increase in fluidity as the DOPC content rises.

The compression modulus profiles in Figure 6b provide further insights into the mechanical properties of these monolayers. Pure DPPC shows a sharp peak, reaching nearly 280 mN/m at an MMA of around 0.45 nm^2^/molecule, indicating a highly condensed and rigid state. In contrast, the compression modulus curves for pure DOPC and DPPC:DOPC mixtures do not exceed 100 mN/m; indicating a more fluid phase throughout the compression range.

The sharp minimum peak at around 5 mN/m is only observed for DPPC. The addition of DOPC diminishes this sharp minimum, disrupting the ordered structure of the DPPC monolayer. Furthermore, the maximum modulus values drop below 100 mN/m, reflecting the increasing dominance of the fluid phase. Additionally, the inset plot of Cs^−1^ versus surface pressure (π) shows that the mixtures do not exhibit the sharp transitions observed in pure DPPC. This highlights the reduced capacity of the mixed monolayer to form a condensed phase. Such behavior is characteristic of biomembranes where unsaturated lipids enhance membrane fluidity and adaptability.

**FIGURE 7:**
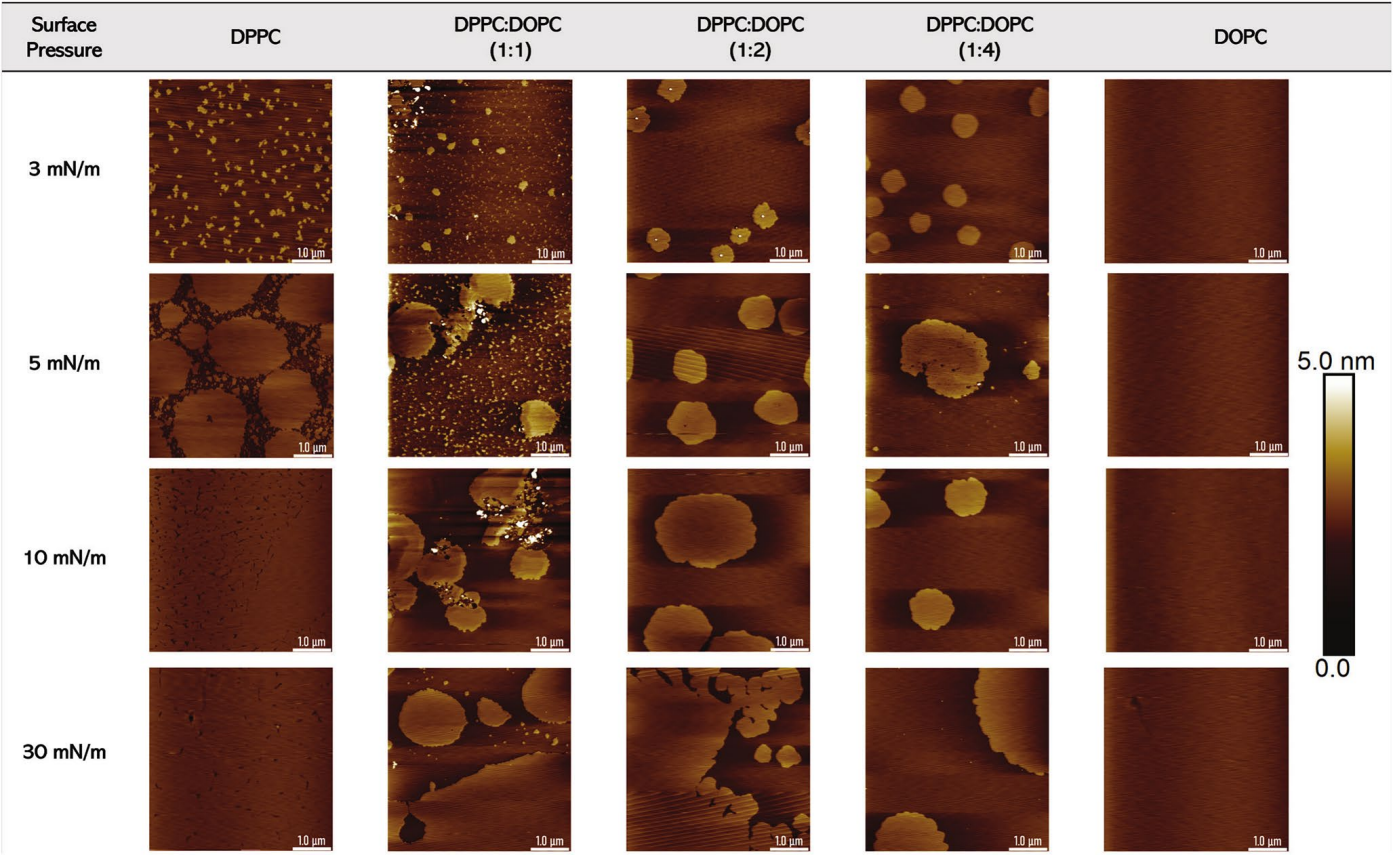
AFM images of lipid mixtures, including DPPC, DPPC‐DOPC (1:1), DPPC‐DOPC (1:2), DPPC‐DOPC (1:4), and DOPC. The lipid deposition was performed at 20°C. The scan area is 5 μm × 5 μm. The white scale bar (at the bottom right) represents 1 μm, while the vertical color bar ranges from 0 to 5 nm.

Figure 7 presents AFM micrographs of monolayer morphologies at surface pressures of 3, 5, 10, and 30 mN/m for various lipid mixtures. The AFM images in the first and last columns correspond to pure DPPC and pure DOPC, respectively. For DPPC, distinct, bright, rounded domains are visible at low surface pressure (3 mN/m), becoming larger, more interconnected, and organized at 5 mN/m. As the pressure increases to 10 mN/m and then to 30 mN/m, the DPPC monolayer transitions to a condensed phase with no isolated lipid domains after lipid domain fusion. In contrast, pure DOPC remains relatively featureless across all pressures, indicating a liquid‐expanded phase.

For the DPPC:DOPC mixtures, the AFM images reveal a gradual change in morphology with increasing DOPC content and surface pressure. At low pressure (3 mN/m), the 1:1 and 1:2 mixtures (second and third columns) display rounded domains similar to those in pure DPPC, but these domains are less densely packed and more widely spaced, resulting in a more heterogeneous phase. The 1:4 mixture (fourth column) at 3 mN/m shows circular lipid domains of a similar size to those in the 1:2 mixture.

As surface pressure increases to 5 and 10 mN/m, the DPPC:DOPC mixtures exhibit significant changes in domain size and distribution. At 5 mN/m, more irregularly shaped domains are observed, especially in the 1:1 and 1:2 mixtures. The higher surface pressure appears to encourage the formation of condensed DPPC regions within a more fluid DOPC matrix. At 10 mN/m, the 1:1 and 1:2 DPPC:DOPC mixtures display fewer but larger domains compared to the 1:4 mixture.

At the highest surface pressure (30 mN/m), the AFM images reveal that DPPC maintains densely packed domains that nearly cover the entire surface. The 1:1 and 1:2 mixtures at this pressure exhibit large, continuous domains with less distinct boundaries, while the 1:4 mixture still displays fluid‐like behavior. Thus, DOPC prevents the formation of highly ordered domains even at high pressures. These observations suggest that DOPC modulates membrane rigidity (and lipid mixing) resulting in a more fluid‐like monolayer.

To further distinguish between the DPPC and DOPC monolayers, we conducted scratching experiments (applying shear forces) and vertical force‐distance curve measurements (see Figure 3). The images from the scratching experiments and the height profile analyses reveal distinct thicknesses for DPPC and DOPC monolayers transferred at a surface pressure of 30 mN/m. The DPPC monolayer exhibits a thickness of about 2.7 nm, whereas the DOPC monolayer is much thinner, with a thickness of approximately 0.5 nm. This difference in thickness reflects the unsaturated acyl chains of DOPC, which create a more loosely packed and fluid structure, preventing the dense packing observed in DPPC.

The force‐distance curves provide further insights into the mechanical properties and thickness of these monolayers. For DPPC, the curve indicates a thickness of approximately 2.5 nm, which is slightly lower than the scratch profile measurement, likely due to tip‐sample interactions. The consistent thickness across both measurements suggests a well‐defined monolayer structure for DPPC. Other studies reported that the thickness of the DPPC monolayer at 30 mN/m is 2.35 nm (evaluated using neutron reflectometry (Carrascosa‐Tejedor et al. 2020)), 2.5 nm (observed using AFM (Oncins et al. 2007)), and 2.39 nm (investigated using multi‐beam interferometry (Kienle et al. 2014)). The breakthrough point aligns with previous studies on force‐distance curves of DPPC monolayers at various temperatures from 22°C to 65°C (Oncins et al. 2007). Specifically, at 22°C, Oncins et al. found that breakthrough occurred at 120 pN; whereas, our breakthrough point was at 190 pN at 20°C. In contrast, the force‐separation curve for DOPC lacks a clear breakthrough point, indicating no distinct mechanical boundary and suggesting a softer, more fluid monolayer that does not resist the indentation force.

## 4 Conclusion

This study highlights the complex interplay of environmental factors and lipid composition in orchestrating the structural and morphological properties of DPPC/DOPC monolayers. Ionic subphases, such as KCl and CaCl_2_, significantly enhance initial surface coverage and domain organization. Divalent ions (Ca^2+^) promote more compact and uniform packing due to stronger ion‐lipid interactions. Temperature strongly influences monolayer behavior; at low temperatures, DPPC achieves higher initial coverage and better domain formation, while elevated temperatures (25°C) reduce packing efficiency and delay domain growth. The addition of DOPC to DPPC alters monolayer organization by increasing fluidity. Higher proportions of DOPC disrupt DPPC's ordered structure, leading to less compact domains. Across all systems, increasing surface pressure enhances surface coverage and transitions from microdomains to compact and continuous films at higher pressures. These findings underscore the critical roles of ionic interactions, temperature, and lipid composition in modulating lipid monolayer properties.

## Author Contributions

**Wisnu Arfian A. Sudjarwo:** conceptualization, investigation, methodology, writing – original draft, formal analysis, data curation, validation. **Jose L. Toca‐Herrera:** conceptualization, funding acquisition, writing – review and editing, supervision, formal analysis.

## Conflicts of Interest

The authors declare no conflicts of interest. Jose L. Toca‐Herrera is a member of the JEMT Editorial Board and co‐author of this article.

## Supporting information


**Data S1.** Supporting Information.
**Figure S1.** Thickness profile of DPPC monolayers deposited at 15°C on subphase water. The vertical color scale ranges from −1 to 1 nm, while the profile thickness ranges from −1.5 to 0.3 nm.
**Figure S2.** Thickness profile of DPPC monolayers deposited at 20°C on subphase water. The vertical color scale ranges from −1 to 2 nm for surface pressures of 3, 5, 20, and 30 mN/m while horizontal color scale for surface pressure of 10 mN/m ranges from −1 to 0.77 nm. The profile thickness ranges from −2 to 0.1 nm.
**Figure S3.** Thickness profile of DPPC monolayers deposited at 25°C on subphase water. The vertical color scale ranges from −1.5 to 1 nm while the profile thickness ranges from −2 to 0.1 nm.

## Data Availability

The data that support the findings of this study are available from the corresponding author upon reasonable request.
