# Supplementary material for: Surface Symphony: Orchestrating DPPC/DOPC Monolayer Behavior
Source: Microsc Res Tech. 2025 Aug 27;89(2):207–17. doi: 10.1002/jemt.70066 (PMC12803605; doi:10.1002/jemt.70066)
Supplement: Supplementary file 1 — Data S1. Supporting Information. Figure S1. Thickness profile of DPPC monolayers deposited at 15°C on subphase water. The vertical color scale ranges from −1 to 1 nm, while the profile thickness ranges from −1.5 to 0.3 nm. Figure S2. Thickness profile of DPPC monolayers deposited at 20°C on subphase water. The vertical color scale ranges from −1 to 2 nm for surface pressures of 3, 5, 20, and 30 mN/m while horizontal color scale for surface pressure of 10 mN/m ranges from −1 to 0.77 nm. The profile thickness ranges from −2 to 0.1 nm. Figure S3. Thickness profile of DPPC monolayers deposited at 25°C on subphase water. The vertical color scale ranges from −1.5 to 1 nm while the profile thickness ranges from −2 to 0.1 nm. [file JEMT-89-207-s001.docx]

**Surface Symphony: Orchestrating DPPC/DOPC monolayer behavior**

Wisnu Arfian A. Sudjarwo^1*^, Jose L. Toca-Herrera^1*^

^1^Institut für Biophysik, Universität für Bodenkultur Wien (BOKU), 1190 Vienna, Austria

* Corresponding authors: [wisnu.sudjarwo@boku.ac.at](mailto:wisnu.sudjarwo@boku.ac.at); [jose.toca-herrera@boku.ac.at](mailto:jose.toca-herrera@boku.ac.at)


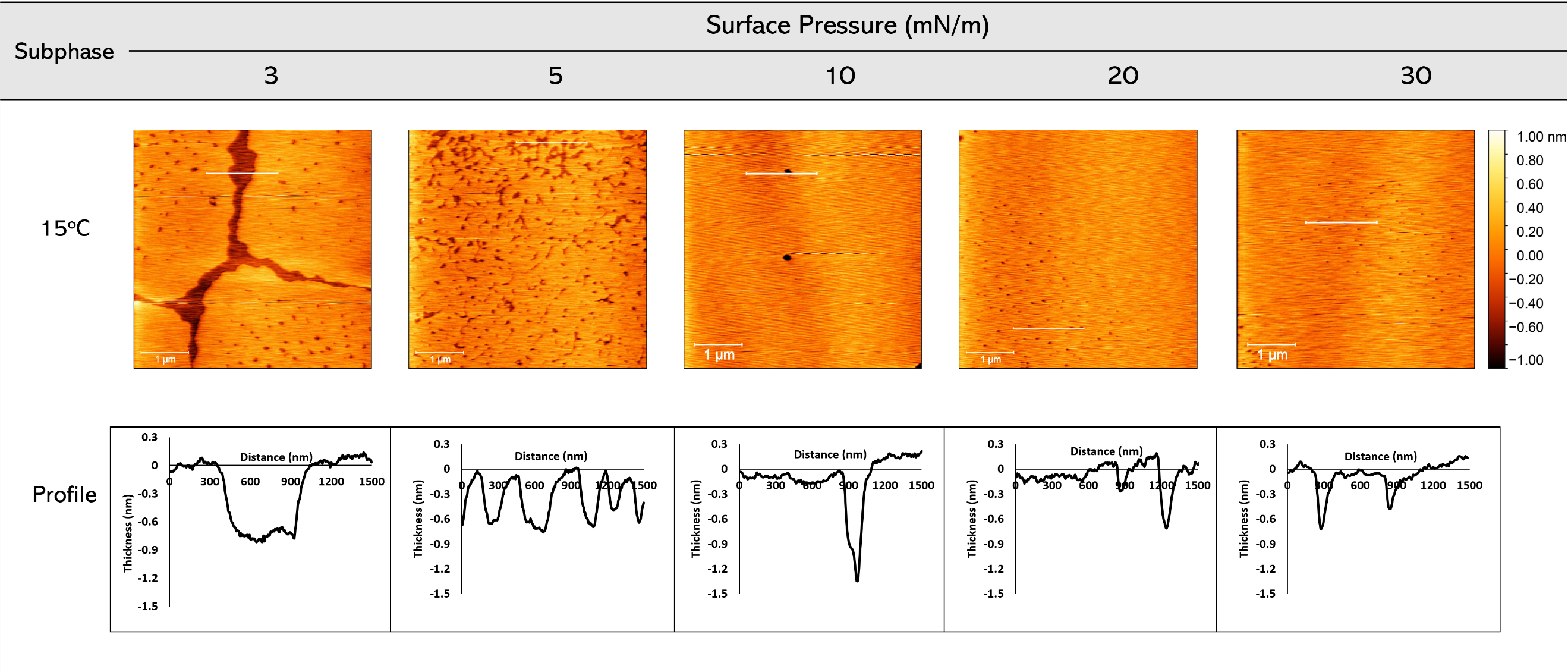


**Figure S1**. Thickness profile of DPPC monolayers deposited at 15^o^C on subphase water. The vertical color scale ranges from -1 to 1 nm, while the profile thickness ranges from -1.5 to 0.3 nm.


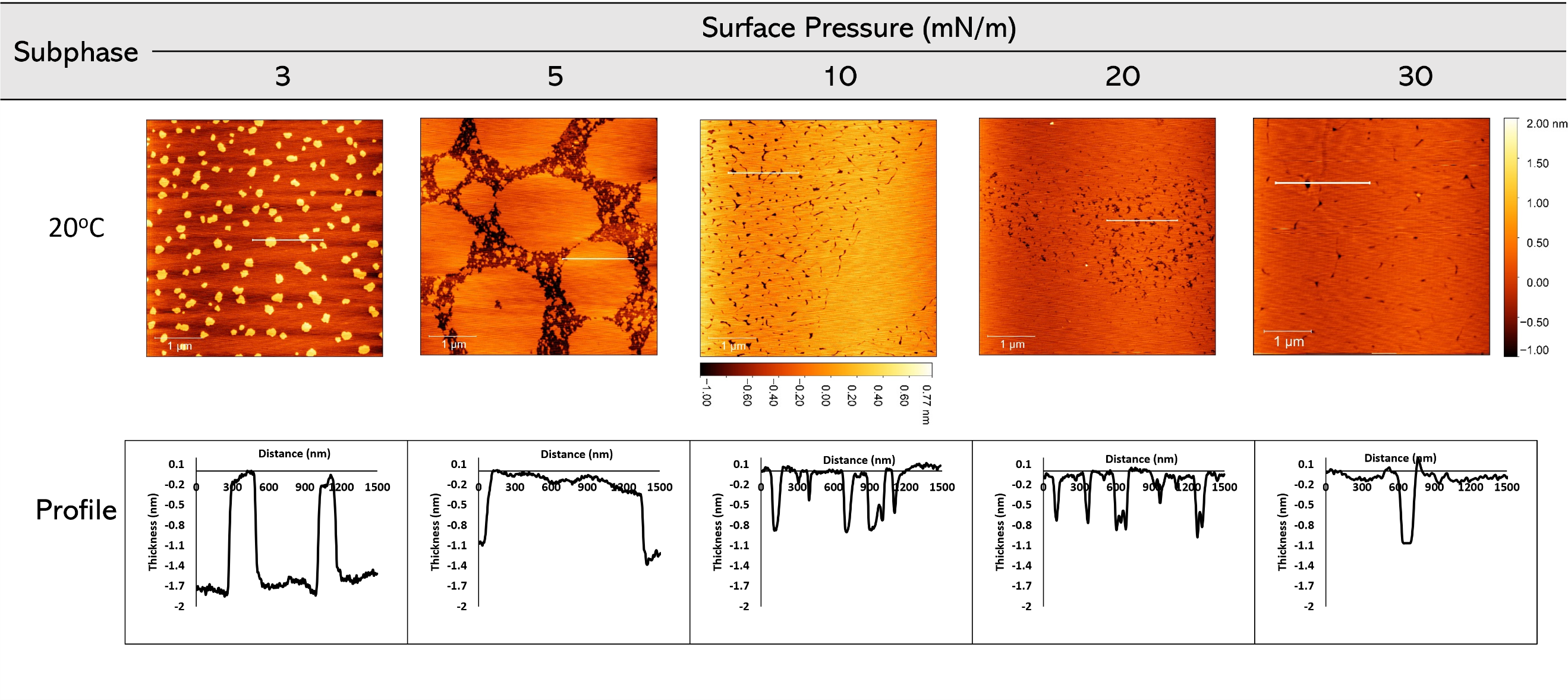


**Figure S2**. Thickness profile of DPPC monolayers deposited at 20^o^C on subphase water. The vertical color scale ranges from -1 to 2 nm for surface pressures of 3 mN/m, 5 mN/m, 20 mN/m, and 30 mN/m while horizontal color scale for surface pressure of 10 mN/m ranges from -1 to 0.77 nm. The profile thickness ranges from -2 to 0.1 nm.


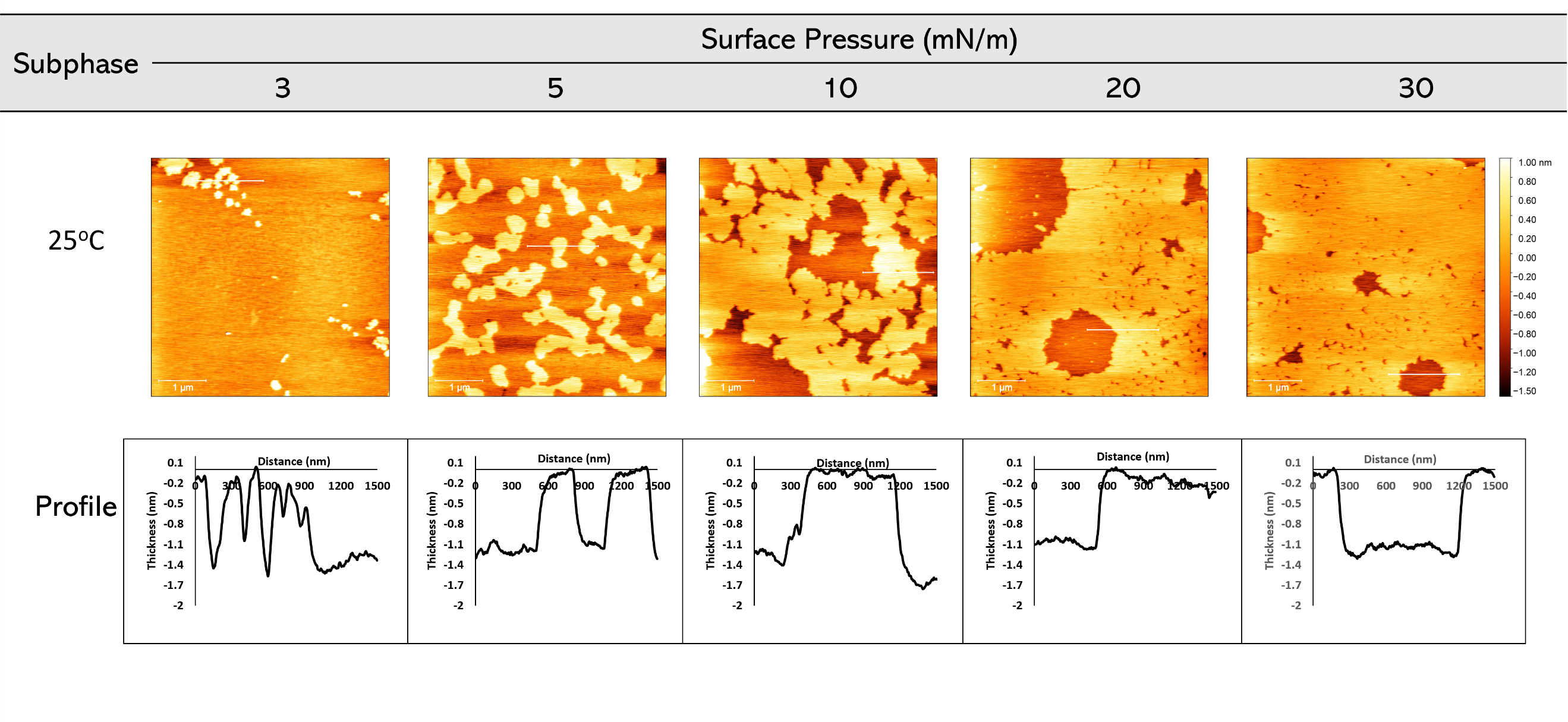


**Figure S3**. Thickness profile of DPPC monolayers deposited at 25^o^C on subphase water. The vertical color scale ranges from -1.5 to 1 nm while the profile thickness ranges from -2 to 0.1 nm.

**Calculation of the applied shear force**

The following equations were used

F = k.δ

δ = defl. sensitivity × (max. defl. setpoint – defl. setpoint)

Where F is force applied (nN), k is spring constant (N/m), and δ is displacement (nm).

Example calculation:

Deflection sensitivity: 36.422 nm/V = 36.422 × 10^-9^ m/V

Spring constant: 0.12 N/m

Deflection until retracted: −1.7 V

Deflection setpoint: 0 V

F = 0.12 N/m × 36.422 × 10^-9^ m/V × (-1.7-0) V

F = -7.437 × 10^-9^ N = -7.437 nN

(The negative sign indicates the direction of the force — i.e., toward the sample — but the magnitude of the applied force is **7.44 nN**.)
